# Estimated Cost-effectiveness of Medical Therapy, Sleeve Gastrectomy, and Gastric Bypass in Patients With Severe Obesity and Type 2 Diabetes

**DOI:** 10.1001/jamanetworkopen.2021.48317

**Published:** 2022-02-14

**Authors:** Brianna N. Lauren, Francesca Lim, Abraham Krikhely, Elsie M. Taveras, Jennifer A. Woo Baidal, Brandon K. Bellows, Chin Hur

**Affiliations:** 1 Department of Medicine, Columbia University Irving Medical Center, New York, New York; 2 Department of Surgery, Columbia University Irving Medical Center, New York, New York; 3 Division of General Academic Pediatrics, Massachusetts General Hospital, Boston; 4 Department of Pediatrics, Columbia University Irving Medical Center, New York, New York

## Abstract

**Importance:**

Bariatric surgery is recommended for patients with severe obesity (body mass index ≥40) and type 2 diabetes (T2D). However, the most cost-effective treatment remains unclear and may depend on the patient’s T2D severity.

**Objective:**

To estimate the cost-effectiveness of medical therapy, sleeve gastrectomy (SG), and Roux-en-Y gastric bypass (RYGB) among patients with severe obesity and T2D, stratified by T2D severity.

**Design, Setting, and Participants:**

This economic evaluation used a microsimulation model to project health and cost outcomes of medical therapy, SG, and RYGB over 5 years. Time horizons varied between 10 and 30 years in sensitivity analyses. Model inputs were derived from clinical trials, large cohort studies, national databases, and published literature. Probabilistic sampling of model inputs accounted for parameter uncertainty. Estimates of US adults with severe obesity and T2D were derived from the National Health and Nutrition Examination Survey. Data analysis was performed from January 2020 to August 2021.

**Exposures:**

Medical therapy, SG, and RYGB.

**Main Outcomes and Measures:**

Quality-adjusted life-years (QALYs), costs (in 2020 US dollars), and incremental cost-effectiveness ratios (ICERs) were projected, with future cost and QALYs discounted 3.0% annually. A strategy was deemed cost-effective if the ICER was less than $100 000 per QALY. The preferred strategy resulted in the greatest number of QALYs gained while being cost-effective.

**Results:**

The model simulated 1000 cohorts of 10 000 patients, of whom 16% had mild T2D, 56% had moderate T2D, and 28% had severe T2D at baseline. The mean age of simulated patients was 54.6 years (95% CI, 54.2-55.0 years), 61.6% (95% CI, 60.1%-63.4%) were female, and 65.1% (95% CI, 63.6%-66.7%) were non-Hispanic White. Compared with medical therapy over 5 years, RYGB was associated with the most QALYs gained in the overall population (mean, 0.44 QALY; 95% CI, 0.21-0.86 QALY) and when stratified by baseline T2D severity: mild (mean, 0.59 QALY; 95% CI, 0.35-0.98 QALY), moderate (mean, 0.50 QALY; 95% CI, 0.25-0.88 QALY), and severe (mean, 0.30 QALY; 95% CI, 0.07-0.79 QALY). RYGB was the preferred strategy in the overall population (ICER, $46 877 per QALY; 83.0% probability preferred) and when stratified by baseline T2D severity: mild (ICER, $36 479 per QALY; 73.7% probability preferred), moderate (ICER, $37 056 per QALY; 85.6% probability preferred), and severe (ICER, $98 940 per QALY; 40.2% probability preferred). The cost-effectiveness of RYGB improved over a longer time horizon.

**Conclusions and Relevance:**

These findings suggest that the effectiveness and cost-effectiveness of bariatric surgery vary by baseline severity of T2D. Over a 5-year time horizon, RYGB is projected to be the preferred treatment strategy for patients with severe obesity regardless of baseline T2D severity.

## Key Points

**Question:**

Compared with medical therapy, is bariatric surgery with sleeve gastrectomy or Roux-en-Y gastric bypass (RYGB) associated with cost-effective weight reduction in patients with severe obesity and varying type 2 diabetes (T2D) severity?

**Findings:**

In this economic evaluation using simulated patient cohorts, RYGB was projected to be the preferred strategy in the overall population with T2D at 5 years (probability preferred, 83.0%). The cost-effectiveness of RYGB was highest in those with mild-to-moderate T2D at baseline.

**Meaning:**

These findings suggest that RYGB is projected to be cost-effective in patients with severe obesity and T2D, regardless of T2D severity.

## Teaser

This economic evaluation estimates the cost-effectiveness of medical therapy, sleeve gastrectomy, and Roux-en-Y gastric bypass among patients with severe obesity and type 2 diabetes, stratified by diabetes severity.

## Introduction

In the US, 15.5% of adults with diabetes, approximately 5.3 million individuals, have severe obesity (body mass index [BMI; weight in kilograms divided by height in meters squared] ≥40.0).^1^ Bariatric surgery is recommended by the American Diabetes Association and other organizations for patients with type 2 diabetes (T2D) and severe obesity.^2^ However, no reference standard procedure for bariatric surgery is well-established, and decision-makers must balance the benefits, risks, and costs of surgery. In the US, sleeve gastrectomy (SG) and Roux-en-Y gastric bypass (RYGB) account for 85% of all primary bariatric surgeries.^3^ Compared with SG, RYGB is associated with greater 5-year total body weight loss (16.1% vs 24.1%; *P* < .001)^4^ and greater T2D remission rates (83.5% vs 86.1%; *P* = .007),^4^ but also is associated with a higher risk of surgical complications.^4,5,6,7,8,9^

T2D remission rates after bariatric surgery may also vary by T2D severity. Scoring systems for defining T2D severity and estimating surgery outcomes have been developed, including the DiaRem score and the Individualized Metabolic Surgery (IMS) score.^10,11^ According to these scoring systems, approximately 90% of patients with mild T2D experience remission after surgery, but only 2% to 12% of patients with severe T2D experience remission. Knowing a patient’s prognosis after bariatric surgery can guide treatment decisions. Although bariatric surgery is cost-effective in patients with T2D,^12,13,14^ it is unknown whether the cost-effectiveness varies by type of surgery and T2D severity.

The purpose of this economic evaluation was to estimate the direct medical costs, quality-adjusted survival, and cost-effectiveness of medical therapy, SG, and RYGB to treat US adults with severe obesity and T2D over 5 years from a health care sector perspective. We also sought to estimate cost-effectiveness of these treatments in these individuals stratified by T2D severity.

## Methods

### Model Overview

**Figure 1.**
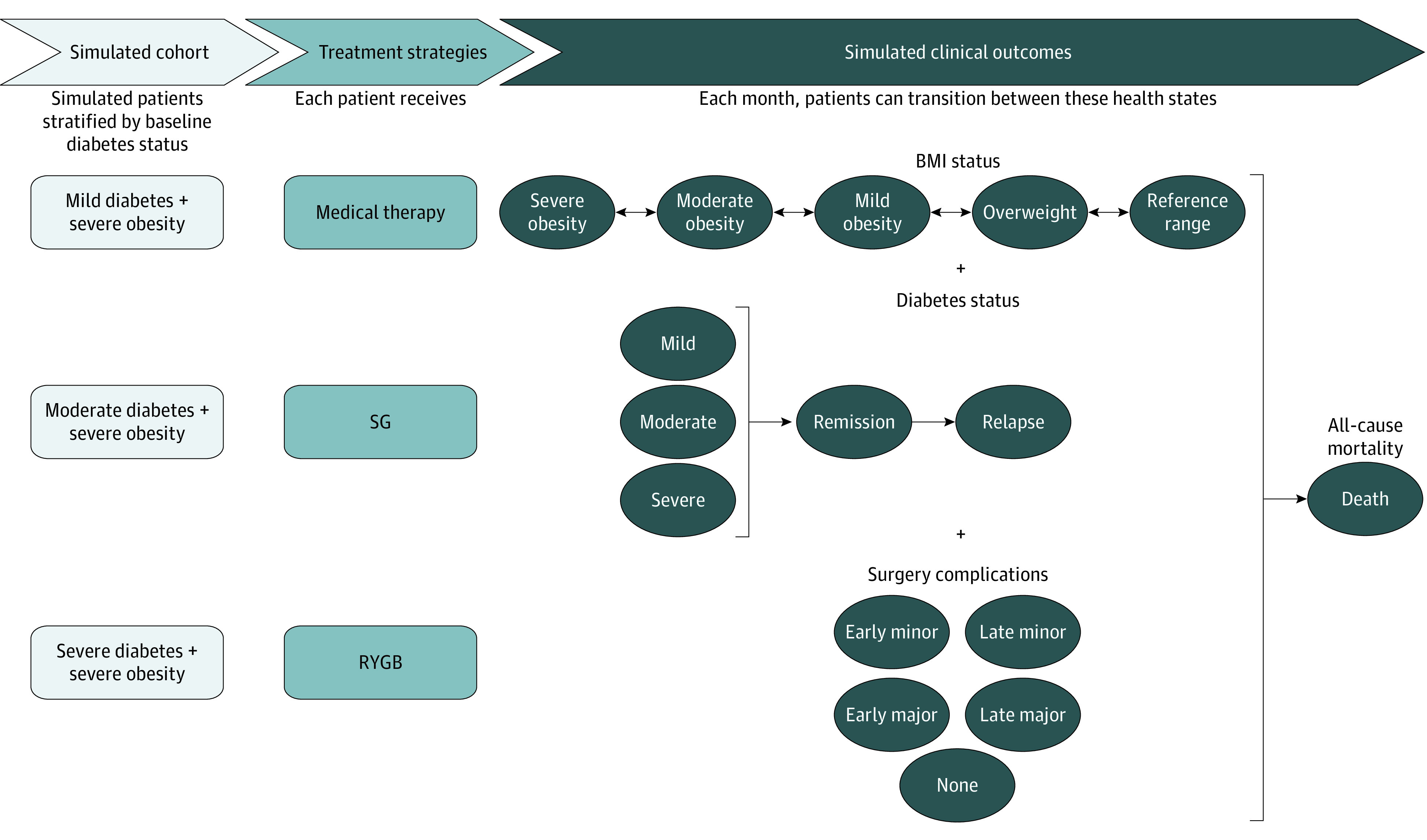
Microsimulation Model Overview BMI indicates body mass index; RYGB, Roux-en-Y gastric bypass; SG, sleeve gastrectomy.

We constructed a patient-level (ie, microsimulation), state-transition model to estimate BMI changes, T2D remission, surgical complications, survival, direct medical costs, and quality of life with medical therapy, SG, and RYGB. Every month of the simulation, patients could transition among health states defined by BMI status (normal, 18-24.9; overweight, 25-29.9; mild obesity, 30-34.9; moderate obesity, 35-39.9; and severe obesity, ≥40), T2D status (baseline severity, remission, and relapse to baseline severity), surgery complications, and mortality (Figure 1). Model inputs were derived from clinical trials, large cohort studies, national databases, and published literature^4,6,7,11,15,16,17,18,19,20,21,22,23,24,25^ (Table 1). This study was exempted from institutional review board approval and informed consent because it is not considered human participants research at Columbia University. Reporting of this study followed the Consolidated Health Economic Evaluation Reporting Standards (CHEERS) reporting guideline.^26^

**Table 1.** Microsimulation Model Inputs

Parameter and treatment	Mean (SE) [range]	Distribution	Source
Total body weight loss, %			
1 y			
Medical therapy	5.0 (3.9) [−2.5 to 12.8]	β	Schauer et al,^6^ 2017
RYGB	29.1 (0.1) [28.8 to 29.3]	β	McTigue et al,^4^ 2020
SG	22.8 (0.2) [22.5 to 23.1]	β	McTigue et al,^4^ 2020
5 y			
Medical therapy	5.0 (5.2) [−5.2 to 15.3]	β	Schauer et al,^6^ 2017
RYGB	24.1 (0.4) [23.3 to 25.0]	β	McTigue et al,^4^ 2020
SG	17.3 (0.6) [14.8 to 17.3]	β	McTigue et al,^4^ 2020
T2D remission according to T2D severity at baseline, %			
Mild			
RYGB	92.8 (3.1) [88.0 to 100.0]	β	Aminian et al,^11^ 2017; Chen et al,^15^ 2018; An et al,^16^ 2019; Aminian,^17^ 2020
SG	85.2 (6.6) [74.0 to 100.0]	β	
Moderate			
RYGB	66.3 (9.4) [60.0 to 97.0]	β	
SG	47.2 (11.0) [25.0 to 68.0]	β	
Severe			
RYGB	12.8 (5.4) [6.0 to 27.0]	β	
SG	6.2 (3.1) [0.0 to 12.0]	β	
T2D relapse by time after remission, %			
1 y			
RYGB	8.4 (0.5) [7.4 to 9.3]	β	McTigue et al,^4^ 2020
SG	11.0 (0.7) [9.6 to 12.4]	β	
3 y			
RYGB	21.2 (1.0) [19.1 to 23.2]	β	
SG	27.2 (1.5) [24.1 to 30.1]	β	
5 y			
RYGB	33.1 (1.8) [29.6 to 36.5]	β	
SG	41.6 (2.4) [36.8 to 46.1]	β	
Surgery complications, %			
30-d mortality			
RYGB	0.2 (0.03) [0.1 to 0.2]	β	Young et al,^19^ 2015
SG	0.1 (0.05) [0.0 to 0.3]	β	
Early complications (1 mo)			
Minor			
RYGB	17.1 (3.1) [11.4 to 23.5]	β	
SG	7.4 (4.3) [5.3 to 22.2]	β	
Major			
RYGB	9.4 (2.4) [5.3 to 14.9]	β	
SG	5.8 (0.9) [9.0 to 12.5]	β	
Late complications (5 y)			
Minor			
RYGB	10.9 (2.4) [4.6 to 14.1]	β	Salminen et al,^7^ 2018
SG	10.7 (2.6) [2.6 to 12.7]	β	
Major			
RYGB	15.1 (1.6) [12.0 to 18.3]	β	
SG	8.3 (1.0) [6.3 to 10.4]	β	
Utilities			
Initial utility, all	0.739 (0.005) [0.729 to 0.749]	β	Sullivan et al,^20^ 2008
Surgery (applied for 6 wk), RYGB and SG	−0.220 (0.010) [−0.240 to −0.220]	β	Campbell et al,^18^ 2010; Klebanoff et al,^25^ 2017
Complications			
Minor (applied for 4 wk), RYGB and SG	−0.110 (0.005) [−0.120 to −0.100]	β	Campbell et al,^18^ 2010; Klebanoff et al,^25^ 2017
Major (applied for 6 wk), RYGB and SG	−0.360 (0.020) [−0.400 to −0.320]	β	Campbell et al,^18^ 2010; Klebanoff et al,^25^ 2017
1 Unit of body mass index decrease, all	0.006 (0.004) [0.000 to 0.017]	β	Hoerger,^21^ 2019; Dennett et al,^22^ 2008; Klebanoff et al,^25^ 2017
Diabetes remission, all	0.110 (0.015) [0.080 to 0.140]	β	Sullivan et al,^20^ 2008
Costs, 2020 US dollars			
Initial surgery			
RYGB	25 070 (4781) [15 699 to 34 442]	γ	Bairdain et al,^23^ 2015; Nguyen et al,^24^ 2013; Klebanoff et al,^25^ 2017
SG	23 708 (5422) [13 081 to 34 334]	γ	
Early complications			
Minor, RYGB and SG	1162 (1778) [813 to 1511]	γ	Campbell et al,^18^ 2010
Major, RYGB and SG	37 881 (5798) [26 517 to 49 245]	γ	
Late complications			
Minor, RYGB and SG	728 (111) [510 to 946]	γ	
Major, RYGB and SG	41 708 (6384) [29 196 to 54 220]	γ	
Healthcare costs, all	Stratified by age, sex, body mass index, and T2D^a^	γ	

Abbreviations: RYGB, Roux-en-Y gastric bypass; SG, sleeve gastrectomy; T2D, type 2 diabetes.

^a^ See eTable 1 and eTable 2 in the Supplement.

### Modeled Patient Populations

The model simulated nationally representative cohorts of US adults (aged ≥18 years) with BMI greater than or equal to 40 and T2D derived from the 1999 to 2018 cycles of the National Health and Nutrition Examination Survey (NHANES). We assumed that patients did not have gastroesophageal reflux disease at baseline (ie, at the time of treatment) because it can be an important factor in bariatric surgery selection.^27^ We identified participants with T2D by self-reported diagnosis, hemoglobin A1_c_ (HbA1_c_) value greater than or equal to 6.5% (to convert to proportion of total hemoglobin, multiply by 0.01), or diabetes medication usage. The IMS score categorized each participant as having mild, moderate, or severe T2D at baseline (eAppendix in the Supplement).^11^ Race and ethnicity categories defined by NHANES were included to simulate our nationally representative cohort. We required individuals to have complete data on modeled characteristics. Of 69 132 NHANES adult participants, 860 met our inclusion criteria.

To ensure stable cost-effectiveness estimates, we simulated 1000 cohorts of 10 000 individuals. Model inputs were randomly selected from predefined statistical distributions for each of the 1000 iterations. To examine heterogeneity in cost-effectiveness estimates by T2D severity at baseline, we also separately modeled 1000 cohorts of 10 000 individuals within each T2D severity subgroup.

### Comparators

We simulated 3 comparators: medical therapy, SG, and RYGB. Medical therapy consisted of lifestyle counseling, weight management, glucose monitoring, and drug therapies, defined by the American Diabetes Association and the Surgical Treatment and Medications Potentially Eradicate Diabetes Efficiently (STAMPEDE) trial protocol.^5,28^ Bariatric procedures were assumed to be performed laparoscopically. SG was defined as the resection of 75% to 80% of the stomach, leaving behind a small gastric tube. RYGB was defined as the creation of a small gastric pouch that connected to the small intestine with Roux-en-Y configuration.^5,7,8,9^

### Weight Loss and T2D Remission

Weight loss and regain with medical therapy were derived from the STAMPEDE trial.^5,6^ We projected weight loss after SG and RYGB surgery using data from the National Patient-Centered Clinical Research Network (PCORNet) Bariatric Study, which reported total body weight loss over 5 years.^4^ We used linear interpolation to estimate monthly weight loss from baseline to 1 year, and partial weight regain from 1 to 5 years. To project BMI changes beyond 5 years in sensitivity analyses, we used the change in BMI by year of age from obese participants (BMI ≥30) in 6 pooled US epidemiological cohort studies from the National Heart, Lung, and Blood Institute Pooled Cohort Study for whom lifetime BMI trajectories were previously developed.^29,30,31,32^

We defined T2D remission as an HbA1_c_ less than 6.5% without diabetes medication. Patients with medical therapy alone were assumed to not achieve remission. The probability of experiencing T2D remission after bariatric surgery was calculated as the weighted average of published T2D remission rates by baseline T2D severity using the IMS scoring system.^11,15,16,17^ We assumed remission occurred at 3 months on the basis of the change in HbA1_c_ levels observed in the STAMPEDE trial.^5^ Patients who experienced T2D remission could subsequently experience a relapse, derived from the PCORNet Bariatric Study.^4^ We assumed that relapse rates were equivalent regardless of baseline T2D severity and that patients who experienced a T2D relapse could not have a second remission. In long-term sensitivity analyses, we assumed a reduction in T2D relapse rates by 50% after 5 years.

### Complications and Mortality

We included the risk of minor and major complications with SG and RYGB, occurring either early (ie, within 1 month of surgery) or late (ie, occurring more than 1 month after surgery). Complication rates were estimated from a large randomized clinical trial comparing SG and RYGB.^7^ We assumed the risk of late complication was constant for the first 4 years, reduced by 50% in years 5 to 10, and 0 after 10 years.^18^ Patients were also at risk of surgery-related mortality within the first month.^19^ Afterward, all-cause mortality rates were estimated using BMI-specific life tables.^33^ Because of the limited data, mortality rates after age 85 years were obtained from 2017 US life tables and assumed to be constant across BMI.^34^

### Quality-of-Life Adjustments and Costs

We assessed the association of treatment with quality of life using utility values, which vary between 0, representing death, and 1, representing perfect health. We derived the baseline utility of T2D and severe obesity, and increase in utility with T2D remission from published literature.^20^ We included an increase in utility per 1-unit reduction in BMI and incorporated short-term decreases in utility associated with surgery and both minor and major surgery complications.^18,21,22^

For patients receiving SG or RYGB, we derived the costs of surgery and complications from previous cost-effectiveness analyses.^18,23,24^ We estimated all other annual total direct health care costs for age, sex, T2D status, and BMI groups using national data (eAppendix, eTable 1, and eTable 2 in the Supplement).^35,36,37,38,39,40,41,42^ All costs were adjusted to 2020 US dollars using the health care component of the Personal Consumption Expenditures price index.^43^

### Validation

We compared the proportion of modeled patients with T2D remission at 3 months with data from the IMS scoring system and total body weight loss over 5 years to data from the National PCORNet Bariatric Study.^4,11^ We compared the overall mean across simulations, the 95% credible interval (ie, 2.5th to 97.5th percentiles), proportion of simulations within the a priori validation target, and visual inspection of the simulated changes over time with the target.

### Statistical Analysis

Our analysis followed recommendations from the Second Panel on Cost-Effectiveness in Health and Medicine (eTable 3 and eTable 4 in the Supplement).^44^ All analyses were performed using Python statistical software version 3.6.5 (Python Software Foundation). Data analysis was performed from January 2020 to August 2021.

Our primary end points were mean direct medical costs in 2020 US dollars, mean quality-adjusted life years (QALYs), and incremental cost-effectiveness ratios (ICERs) from a health care sector perspective (ie, including all direct medical costs regardless of payer). All future costs and QALYs were discounted 3% annually. Costs were disaggregated as intervention costs (stratified as surgery and surgery complications) and all other health care costs (stratified as diabetes, obesity, and any other background costs). Secondary end points included proportion achieving T2D remission and mean BMI at 5 years.

Our primary analyses estimated the cost-effectiveness of medical therapy, SG, and RYGB over 5 years. The means and 95% credible intervals for direct medical costs, QALYs, and all secondary outcomes were calculated across the 1000 simulated iterations for the overall population and within each subgroup of baseline T2D severity. A strategy was considered cost-effective if the ICER was less than $100 000 per QALY gained and the preferred strategy resulted in the greatest number of QALYs gained while being cost-effective.^45^

Deterministic 1-way sensitivity analyses estimated the impact of independently varying each parameter across a plausible range while keeping all other inputs constant at their mean value (eAppendix in the Supplement). The upper and lower bounds for each parameter were obtained from literature or calculated from 95% CIs. One-way sensitivity analyses were performed for the overall population and each subgroup of baseline T2D severity using 1 cohort of 10 000 individuals over 5 years. We also performed sensitivity analysis examining cost-effectiveness over 10- and 30-year time horizons.

## Results

### Modeled Population

The model simulated 1000 cohorts of 10 000 patients, of whom 16% had mild T2D, 56% had moderate T2D, and 28% had severe T2D at baseline. The mean age of simulated patients was 54.6 years (95% CI, 54.2-55.0 years), 61.6% (95% CI, 60.1%-63.4%) were female, 65.1% (95% CI, 63.6%-66.7%) were non-Hispanic White, and mean BMI was 45.8 (95% CI, 45.7-46.0) (eTable 5 in the Supplement). Patients had a mean HbA1_c_ of 7.4% (95% CI, 7.4%-7.5%), 31.0% (29.4%-32.3%) were using insulin, 77.6% (95% CI, 76.3%-78.8%) were using an oral diabetes medication, and the majority had moderate T2D (55.8%; 95% CI, 54.5%-57.2%).

### Model Validation and Projected Clinical Outcomes

The model replicated validation targets for the proportion of patients achieving weight loss changes and T2D remission at 1 and 5 years (eFigure 1, eFigure 2, and eTable 6 in the Supplement). The projected mean BMI with SG decreased to 35.4 (95% CI, 35.2-35.5) at 1 year, before increasing to 38.5 (95% CI, 37.9-39.0) at 5 years (eFigure 3 in the Supplement). For RYGB, the mean BMI was 32.5 (95% CI, 32.3-32.7) at 1 year, before increasing to 34.8 (95% CI, 34.4-35.2) at 5 years. Three months after surgery, the projected proportion of T2D remission was highest in those who underwent RYGB and had mild T2D at baseline (92.9%; 95% CI, 86.4%-97.6%) and lowest in those who underwent SG and had severe diabetes at baseline (5.7%; 95% CI, 1.0%-13.9%).

### Cost-effectiveness Outcomes

**Figure 2.**
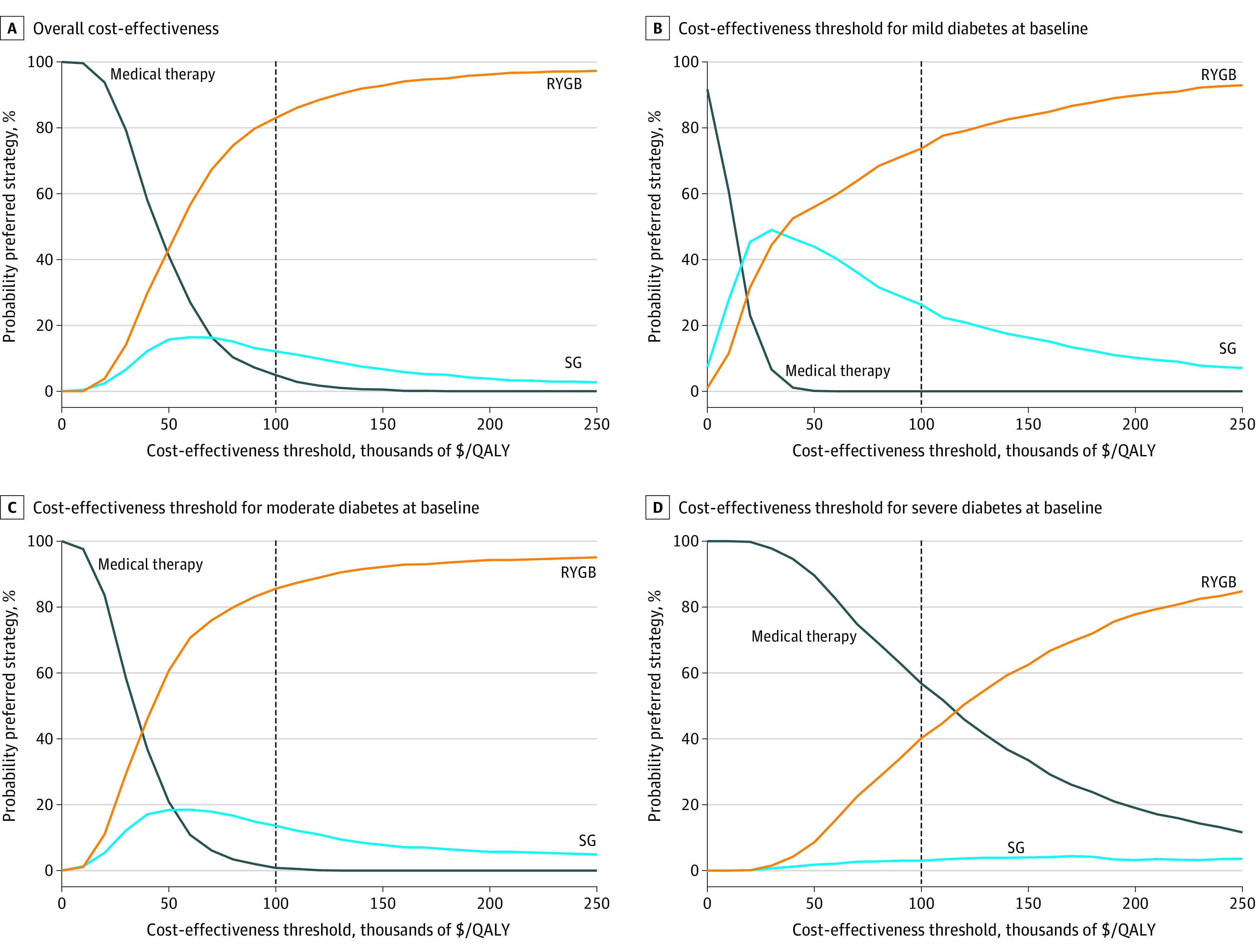
Cost-effectiveness Acceptability Curves Over 5-Year Time Horizon QALY indicates quality-adjusted life-year; RYGB, Roux-en-Y gastric bypass; SG, sleeve gastrectomy.

Compared with medical therapy in the overall population, the model projected SG to increase mean costs per patient by $18 634 (95% CI, $7861-$30 988) and RYGB by $20 633 (95% CI, $10 269-$32 937) over 5 years (Table 2). Compared with medical therapy, SG was projected to increase QALYs by a mean of 0.31 QALY (95% CI, 0.13-0.66 QALY). RYGB gained the most QALYs in the overall population (mean, 0.44 QALY; 95% CI, 0.21-0.86 QALY) and when stratified by baseline T2D severity: mild (mean, 0.59 QALY; 95% CI, 0.35-0.98 QALY), moderate (mean, 0.50 QALY; 95% CI, 0.25-0.88 QALY), and severe (mean, 0.30 QALY; 95% CI, 0.07-0.79 QALY). In the overall population, RYGB was cost-effective vs medical therapy (ICER of $46 877 per QALY gained) with an 83.0% probability of being the preferred strategy (Table 2 and Figure 2). SG was extendedly dominated by RYGB, meaning that it gained fewer QALYs than RYGB at a higher cost per QALY gained, representing a less-efficient use of resources. RYGB remained the preferred strategy when stratifying by baseline T2D severity. Only in patients with mild T2D was SG not extendedly dominated by RYGB. RYGB was the most cost-effective in those with mild T2D at baseline (ICER vs SG $36 479 per QALY gained; 73.7% probability preferred), less so for those with moderate T2D at baseline (ICER, $37 056 per QALY; 85.6% probability preferred), and least cost-effective in those with severe T2D at baseline (ICER vs medical therapy, $98 940 per QALY gained; 40.2% probability preferred) (eFigure 4 in the Supplement).

**Table 2.** Cost-effectiveness Results Over 5-Year Time Horizon^a^

Category	Medical therapy	Sleeve gastrectomy	Roux-en-Y gastric bypass
Overall			
Costs, mean, $	61 620	80 254	82 253
Incremental costs, mean (95% CI), $	1 [Reference]	18 634 (7861 to 30 988)	20 633 (10 269 to 32 937)
QALY, mean	3.33	3.64	3.77
Incremental QALYs, mean (95% CI)	1 [Reference]	0.31 (0.13 to 0.66)	0.44 (0.21 to 0.86)
ICER ($/QALY gained)^b^	1 [Reference]	Extendedly dominated	46 877
Probability preferred strategy, %^c^	4.9	12.1	83.0
Mild T2D at baseline			
Costs, mean, $	58 949	67 244	71 059
Incremental costs, mean (95% CI), $	1 [Reference]	8296 (−2416 to 20 809)	12 111 (2137 to 23 728)
QALY, mean	3.40	3.89	3.99
Incremental QALYs, mean (95% CI)	1 [Reference]	0.49 (0.30 to 0.85)	0.59 (0.35 to 0.98)
ICER ($/QALY gained)^b^	1 [Reference]	16 926	36 479
Probability preferred strategy, %^c^	0.0	26.3	73.7
Moderate T2D at baseline			
Costs, mean, $	61 271	78 550	79 841
Incremental costs, mean (95% CI), $	1 [Reference]	17 279 (5873 to 30 351)	18 570 (7665 to 31 649)
QALY, mean	3.33	3.68	3.83
Incremental QALYs, mean (95% CI)	1 [Reference]	0.35 (0.16 to 0.68)	0.50 (0.25 to 0.88)
ICER ($/QALY gained)^b^	1 [Reference]	Extendedly dominated	37 056
Probability preferred strategy, %^c^	0.8	13.6	85.6
Severe T2D at baseline			
Costs, mean, $	63 848	90 848	93 773
Incremental costs, mean (95% CI), $	1 [Reference]	27 000 (16 754 to 39 870)	29 925 (18 999 to 42 188)
QALY, mean	3.30	3.49	3.60
Incremental QALYs, mean (95% CI)	1 [Reference]	0.20 (0.03 to 0.56)	0.30 (0.07 to 0.79)
ICER ($/QALY gained)^b^	1 [Reference]	Extendedly dominated	98 940
Probability preferred strategy, %^c^	56.8	3.0	40.2

Abbreviations: ICER, incremental cost-effectiveness ratio; QALY, quality-adjusted life-year; T2D, type 2 diabetes.

^a^ All costs are shown in 2020 US dollars.

^b^ ICERs are calculated using the mean costs and QALYs from the 1000 probabilistic iterations and are referent to the next least costly, nondominated strategy. Extendedly dominated indicates that the strategy gains fewer QALYs and costs more per QALY gained than another strategy, representing inefficient use of resources.

^c^ Probability of being the preferred strategy is presented at a cost-effectiveness threshold of $100 000 per QALY gained.

**Figure 3.**
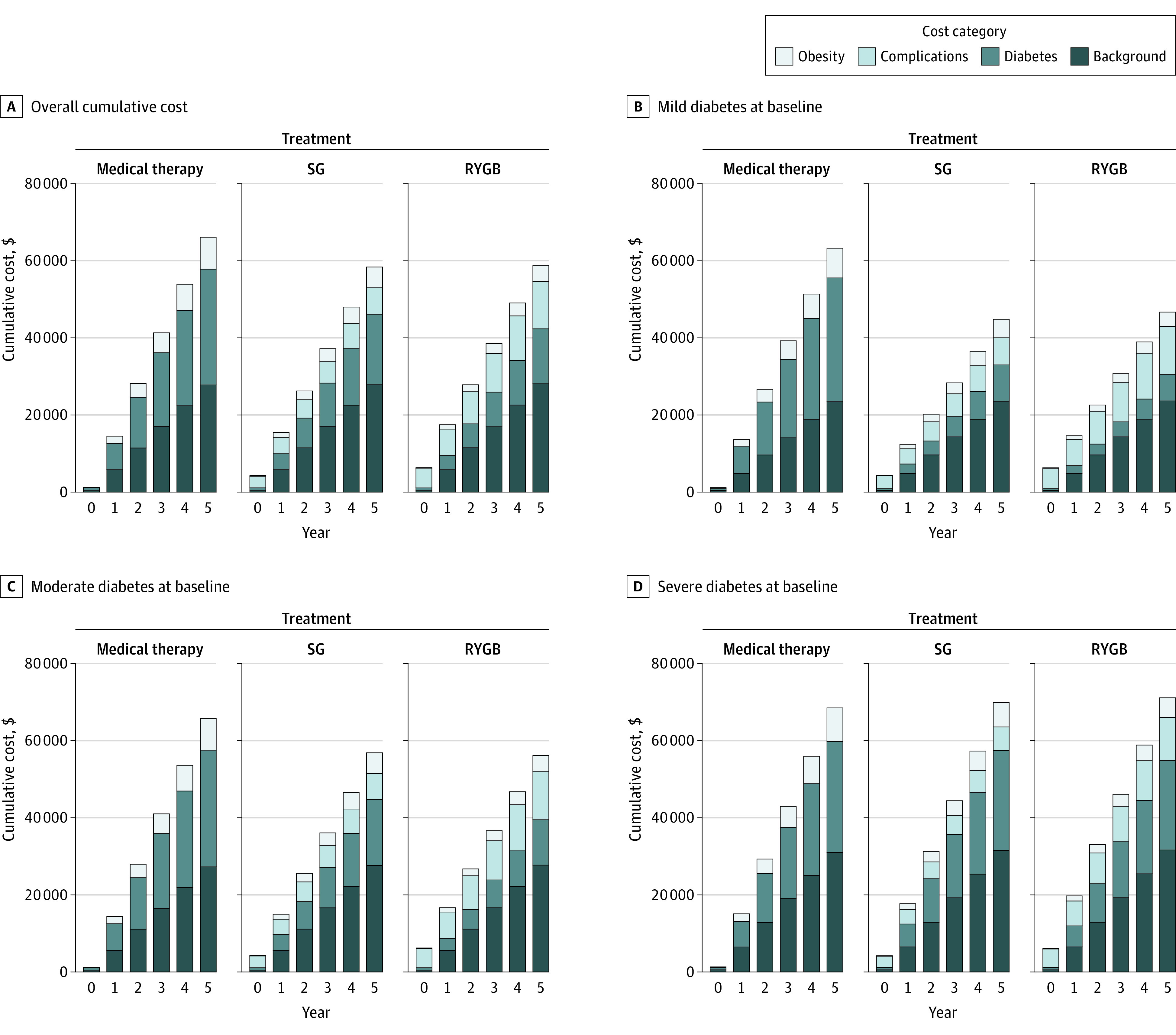
Cumulative Health Care Costs Stratified by Type, Excluding Initial Cost of Surgery RYGB indicates Roux-en-Y gastric bypass; SG, sleeve gastrectomy.

Figure 3 shows the projected cumulative health care costs (excluding bariatric surgery costs) for medical therapy, SG, and RYGB for the overall population and within each T2D severity subgroup. Compared with medical therapy, SG and RYGB reduced health care costs associated with both T2D and obesity but had increased costs associated with complications. However, the reduction in T2D and obesity costs was lower in the severe T2D subgroup, and increased complication costs resulted in higher cumulative costs than that of medical therapy, even when excluding the cost of surgery itself.

### Sensitivity Analyses

Results from 1-way sensitivity analyses showed our model was most sensitive to an increase in utility per 1-unit decrease in BMI, weight loss achieved with medical therapy, and the cost of RYGB surgery (eFigure 5 in the Supplement). RYGB was the preferred strategy at our cost-effectiveness threshold (<$100 000 per QALY) in all 1-way sensitivity analyses in the overall population. However, the preferred strategy switched to SG when the cost of RYGB was the maximum (ie, $34 442) in patients with mild T2D and when the cost of SG was at the minimum (ie, $13 081) in patients with severe T2D. Over 5 years in patients with severe T2D at baseline, there were values for each parameter that resulted in medical therapy being the preferred strategy.

The cost-effectiveness of RYGB increased with longer time horizons (eTable 7 and eTable 8 in the Supplement). RYGB had a 98.1% probability of being the preferred strategy in the overall population over 10 years, with improved cost-effectiveness vs medical therapy of $17 497 per QALY gained (eFigure 6 in the Supplement) and a nearly 100% probability, regardless of baseline T2D severity, over 30 years (eFigure 7 in the Supplement).

## Discussion

In this economic evaluation, we estimated the cost-effectiveness of SG and RYGB bariatric surgery interventions compared with medical therapy over 5 years in US adults with T2D and severe obesity. At a cost-effectiveness threshold of $100 000 per QALY gained, RYGB was projected to be the preferred treatment strategy regardless of baseline T2D severity. We estimated that RYGB would cost $46 877 per QALY gained compared with medical therapy and have an 83.0% probability of being the preferred strategy. However, our findings were sensitive to the severity of T2D. RYGB only had a 40.2% probability of being the preferred strategy in patients with severe T2D at baseline. Overall, the cost-effectiveness of RYGB compared with medical therapy improved to $17 497 per QALY gained when projected over a longer 10-year time horizon and became dominant (ie, cost less and more effective) over a 30-year time horizon.

Although several published analyses^12,13,14,18,25,46^ have demonstrated the cost-effectiveness of bariatric surgery in patients with obesity and T2D, less is known about whether T2D severity is associated with the optimal surgery a patient should undergo. Most studies only included the presence of T2D with severe obesity. However, Hoerger et al^12^ examined the cost-effectiveness of bariatric surgery separately for patients with newly diagnosed T2D and those with established T2D. Although their analysis incorporated a binary classification of T2D duration, it did not include other patient characteristics, such as insulin use, diabetes medication use, and glycemic control, that differentiate T2D response to bariatric surgery. A strength of our analysis is a more comprehensive incorporation of T2D severity using multiple patient characteristics associated with T2D management. To our knowledge, this analysis is the first to compare SG and RYGB in a nationally representative population of patients with severe obesity and T2D and to examine the association of T2D severity with cost-effectiveness. Our analysis underscores the benefit of estimating the heterogeneity of cost-effectiveness estimates across specific patient profiles. Determining which subgroups may benefit the most from a specific strategy can lead to the improved personalization of medical treatment.

RYGB remained the preferred strategy in the majority of sensitivity analyses, emphasizing its effectiveness in treating severe obesity and T2D despite its higher costs and rates of surgical complications. SG became the preferred strategy only when surgical costs were varied in patients with mild or severe T2D at baseline. Over a short time horizon, medical therapy may be preferred in those with severe T2D at baseline because of the high cost of surgery and high rates of complications.^11^ However, the benefits of RYGB, such as increased T2D remission and greater sustained weight loss, were projected to offset these detriments over longer time horizons. This finding is consistent with existing evidence that the cost-effectiveness of bariatric surgery improves with longer time horizons.^13^ In addition, our BMI projections show trends over 20 years similar to those from a recent report from The Swedish Obesity Study, which found that the BMI of surgical patients stabilized at a lower BMI value compared with the baseline.^47^

### Limitations

The results of our analysis should be considered in the context of the following limitations. Microsimulation models can simplify complex processes using biased input data, but we attempted to remedy these concerns by stating our assumptions and validating our results with clinical data. We did not incorporate all types of bariatric surgery as comparators in our model, instead focusing on the most common approaches. We excluded laparoscopic adjustable gastric banding and biliopancreatic diversion with duodenal switch, which are less common procedures within the US.^48^ Laparoscopic adjustable gastric banding is associated with lower weight loss and rates of T2D remission compared with RYGB and SG, whereas biliopancreatic diversion with duodenal switch is associated with greater weight loss and T2D remission with a potentially higher complication rate.^49,50,51^ We did not incorporate reversal for RYGB, but it is performed in only a small number of patients who experience serious complications.^52^ To ensure that patients were eligible for each treatment strategy in our analysis, we assumed that individuals with gastroesophageal reflux disease, which may be worsened by SG and result in a conversion from SG to RYGB, were not included. However, some model parameters may have been derived from populations that included gastroesophageal reflux disease. Further research is needed to understand the impact this may have on the cost-effectiveness of bariatric surgery. In addition, as new drug therapies emerge in the medical treatment of obesity, their effectiveness and cost-effectiveness should be reevaluated in comparison to bariatric surgery.

## Conclusions

In this study, over 5 years, RYGB was projected to result in greater weight loss and T2D remission rates than SG and medical therapy in US adults with severe obesity (BMI ≥40) and T2D, regardless of T2D severity at baseline. Despite its higher upfront surgical costs, RYGB was estimated to be the most cost-effective treatment over 5 years and became even more cost-effective over longer time horizons (eg, 10 and 30 years).

## Notes

Supplement. **eAppendix.** Supplemental Methods **eTable 1.** Age- and Sex-Specific Background Healthcare Costs **eTable 2.** Additive Healthcare Costs Associated with T2DM and Obesity **eTable 3.** Reporting Checklist for Cost-Effectiveness Analyses from the Second Panel on Cost-Effectiveness in Health and Medicine **eTable 4.** Formal Health Care Sector Impact Inventory Assessment **eTable 5.** Weighted NHANES and Baseline Population Characteristics **eTable 6.** T2DM Remission Model Validation **eTable 7.** Cost-Effectiveness Results Over 10-Year Time Horizon **eTable 8.** Cost-Effectiveness Results Over 30-Year Time Horizon **eFigure 1.** Weight Loss Model Validation **eFigure 2.** T2DM Remission Over 5-Year Time Horizon **eFigure 3.** BMI Over 30-Year Time Horizon **eFigure 4.** Incremental Cost-Effectiveness Scatterplot Over 5-Year Time Horizon **eFigure 5.** One-Way Sensitivity Analysis Over 5-Year Time Horizon **eFigure 6.** Cost-Effectiveness Acceptability Curves Over 10-Year Time Horizon **eFigure 7.** Cost-Effectiveness Acceptability Curves Over 30-Year Time Horizon
eReferences
