# Supplementary material for: Estimated Cost-effectiveness of Medical Therapy, Sleeve Gastrectomy, and Gastric Bypass in Patients With Severe Obesity and Type 2 Diabetes
Source: JAMA Netw Open. 2022 Feb 14;5(2):e2148317. doi: 10.1001/jamanetworkopen.2021.48317 (PMC8845022; doi:10.1001/jamanetworkopen.2021.48317)
Supplement: Supplement. — eAppendix. Supplemental Methods eTable 1. Age- and Sex-Specific Background Healthcare Costs eTable 2. Additive Healthcare Costs Associated with T2DM and Obesity eTable 3. Reporting Checklist for Cost-Effectiveness Analyses from the Second Panel on Cost-Effectiveness in Health and Medicine eTable 4. Formal Health Care Sector Impact Inventory Assessment eTable 5. Weighted NHANES and Baseline Population Characteristics eTable 6. T2DM Remission Model Validation eTable 7. Cost-Effectiveness Results Over 10-Year Time Horizon eTable 8. Cost-Effectiveness Results Over 30-Year Time Horizon eFigure 1. Weight Loss Model Validation eFigure 2. T2DM Remission Over 5-Year Time Horizon eFigure 3. BMI Over 30-Year Time Horizon eFigure 4. Incremental Cost-Effectiveness Scatterplot Over 5-Year Time Horizon eFigure 5. One-Way Sensitivity Analysis Over 5-Year Time Horizon eFigure 6. Cost-Effectiveness Acceptability Curves Over 10-Year Time Horizon eFigure 7. Cost-Effectiveness Acceptability Curves Over 30-Year Time Horizon eReferences [file jamanetwopen-e2148317-s001.pdf]

Supplemental Online Content

Lauren BN, Lim F, Krikhely A, et al. Estimated cost-effectiveness of medical therapy, sleeve gastrectomy, and gastric bypass in patients with severe obesity and type 2 diabetes. *JAMA Netw Open*. 2022;5(2):e2148317.
doi:10.1001/jamanetworkopen.2021.48317

eAppendix. Supplemental Methods
eTable 1. Age- and Sex-Specific Background Healthcare Costs
eTable 2. Additive Healthcare Costs Associated with T2DM and Obesity
eTable 3. Reporting Checklist for Cost-Effectiveness Analyses from the Second Panel on Cost-Effectiveness in Health and Medicine
eTable 4. Formal Health Care Sector Impact Inventory Assessment
eTable 5. Weighted NHANES and Baseline Population Characteristics
eTable 6. T2DM Remission Model Validation
eTable 7. Cost-Effectiveness Results Over 10-Year Time Horizon
eTable 8. Cost-Effectiveness Results Over 30-Year Time Horizon
eFigure 1. Weight Loss Model Validation
eFigure 2. T2DM Remission Over 5-Year Time Horizon
eFigure 3. BMI Over 30-Year Time Horizon
eFigure 4. Incremental Cost-Effectiveness Scatterplot Over 5-Year Time Horizon
eFigure 5. One-Way Sensitivity Analysis Over 5-Year Time Horizon
eFigure 6. Cost-Effectiveness Acceptability Curves Over 10-Year Time Horizon
eFigure 7. Cost-Effectiveness Acceptability Curves Over 30-Year Time Horizon
eReferences

This supplemental material has been provided by the authors to give readers additional information about their work.

© 2022 Lauren BN et al. *JAMA Network Open.*

eAppendix. Supplemental Methods

Individualized Metabolic Surgery (IMS) Score Calculation

The following equation, developed by Aminian et al (2017)[1], was used to calculate an Individualized Metabolic Surgery (IMS) Score for each modeled individual in our simulation. It considers number of diabetes medication, insulin use, duration of type 2 diabetes mellitus (T2DM), and glycemic control (HbA1c) before surgical intervention (preop). This score was then used to divide patients into three different levels of type 2 diabetes mellitus (T2DM) severity: mild (IMS score ≤ 25), moderate (25 < IMS score ≤ 95), and severe (IMS score > 95). Patients with mild T2DM are more likely to have good glycemic control and less likely to be on insulin before surgery compared to other severity levels. Patients with higher severity of T2DM are more likely to be on insulin and have a high number of diabetes medication with poor glycemic control as well as a long duration of T2DM history.

$$\begin{gathered} IMS = 12.6 * (preop\ number\ of\ T2DM\ medications) + 18 * (preop\ insulin\ use) + 16 \\ * (1 - preop\ glycemic\ control) + I_{(0<preop\ duration\ of\ T2DM \le 5)} * 5.6 \\ * (preop\ duration\ of\ T2DM) + I_{(5<preop\ duration\ of\ T2DM \le 10)} \\ * [28 + 4 * (preop\ duration\ of\ T2DM - 5)] + I_{(10<preop\ duration\ of\ T2DM \le 15)} \\ * [48 + 2 * (preop\ duration\ of\ T2DM - 10)] + I_{(15<preop\ duration\ of\ T2DM \le 40)} \\ * [58 + 1.68 * (preop\ duration\ of\ T2DM - 15)] \end{gathered}$$

Where the following are indicator variables:

$$preop\ insulin\ use = \begin{cases} 1, & if\ preop\ insulin\ use = Yes \\ 0, & if\ preop\ insulin\ use = No \end{cases}$$

$$preop\ glycemic\ control = \begin{cases} 1, & if\ preop\ glycemic\ control = Yes \\ 0, & if\ preop\ glycemic\ control = No \end{cases}$$

$$I_{(0<preop\ duration\ of\ T2DM \le 5)} = \begin{cases} 1, & if\ 0\ years < preop\ duration\ of\ T2DM \le 5\ years \\ 0, & if\ preop\ duration\ of\ T2DM > 5\ years \end{cases}$$

$$I_{(5<preop\ duration\ of\ T2DM \le 10)} = \begin{cases} 1, & if\ 5\ years < preop\ duration\ of\ T2DM \le 10\ years \\ 0, & if\ preop\ duration\ of\ T2DM \le 5\ years\ or\ > 10\ years \end{cases}$$

$$I_{(10<preop\ duration\ of\ T2DM \le 15)} = \begin{cases} 1, & if\ 10\ years < preop\ duration\ of\ T2DM \le 15\ years \\ 0, & if\ preop\ duration\ of\ T2DM \le 10\ years\ or > 15\ years \end{cases}$$

$$I_{(15<preop\ duration\ of\ T2DM \le 40)} = \begin{cases} 1, & if\ 15\ years < preop\ duration\ of\ T2DM \le 40\ years \\ 0, & if\ preop\ duration\ of\ T2DM \le 15\ years \end{cases}$$

Healthcare Costs

As in prior analyses, we estimated the total healthcare expenditures of United States (U.S.) adults from U.S. Medical Expenditure Panel Survey (MEPS) using a two-part model.[2–4] First, we used a survey-weighted multivariable logistic regression model to estimate the probability of non-zero total annual healthcare expenditures. Second, among individuals with non-zero total healthcare expenditures, we used a survey-weighted multivariable generalized linear model with a log link and gamma distribution to estimate annual healthcare costs. We included the same covariates in the generalized linear model as in the logistic regression.

As MEPS does not include institutionalized individuals receiving long-term care, we separately estimated the mean, weighted cost of long-term care, stratified by age and sex.[2] We divided the number of U.S. adults using long-term care in 2013-2014 (i.e., using adult day service centers or residing in nursing homes or assisted living facilities), stratified by age (i.e., <65, 65-74, 75-84, ≥85) and sex, by the number of U.S. adults in the same age and sex strata in the 2010 U.S. Census.[5,6] We multiplied the proportion of all U.S. adults using each type of long-term care service by published annual long-term care cost estimates from the US Department of Health and Human Services and the Genworth Cost of Care Survey, to estimate the mean, weighted cost of long-term care.[7,8]

We used the combined two-part model to estimate mean costs and standard errors for age (18-34, 35-44, 45-54, 55-64, 65-74, 75-84, and ≥85 years), sex, T2DM (yes or no), and BMI (18-24.9, 25-29.9, 30-34.9, 35-39.9, and ≥40 kg/m2) stratified groups. Total background healthcare costs (including long-term care costs) for age- and

© 2022 Lauren BN et al. *JAMA Network Open.*

sex-groups are shown in **eTable 1**. Total background healthcare costs represent all non-diabetes, non-overweight/obesity, and non-surgery related healthcare costs. The age-specific costs of having diabetes or overweight/obesity are shown in **eTable 2**. During each year of the simulation, total healthcare costs are calculated by adding the total background healthcare costs, costs of diabetes, costs of overweight/obesity, and costs of surgery and surgical complications.

Net Monetary Benefit (NMB)

Net monetary benefit (NMB) is the monetary value of an intervention for a given cost-effectiveness (CE) threshold. Incremental net monetary benefit (INMB) is calculated as the incremental effectiveness (in quality-adjusted life years, QALYs) of a strategy multiplied by the WTP and then subtracted by its incremental costs.

$$INMB = \Delta QALYs * CE\ Threshold - \Delta Costs$$

An INMB >0 indicates the intervention is cost-effective compared to the reference strategy for the given CE threshold, while an INMB ≤0 indicates that the reference strategy is preferred. Additionally, the greater the INMB, the more cost-effective the strategy is compared with the reference strategy. When more than two interventions are considered simultaneously, the strategy with the highest INMB is considered the preferred strategy (i.e., the most cost-effective).

The results of deterministic one-way sensitivity analysis, where each model input is varied across a plausible range while all others are held constant, are generally visualized with a tornado diagram. The variable with the largest effect on INMB is located at the top, while the variable with the smallest effect is shown at the bottom. Horizontal bars represent the range of INMB resulting from changes in the model input. Changes in preferred strategy are indicated with a colored vertical bar at the INMB value where the change in preferred strategy occurs.

© 2022 Lauren BN et al. *JAMA Network Open.*

eTable 1. Age- and Sex-Specific Background Healthcare Costs

Age	Mean	SE
Males		
18-34	$2,367.07	$12.70
35-44	$3,308.56	$23.71
45-54	$4,217.39	$26.91
55-64	$5,419.60	$36.60
65-74	$8,604.11	$63.05
75-84	$12,732.95	$78.53
85+	$23,458.68	$78.53
Females		
18-34	$2,451.61	$12.70
35-44	$3,393.10	$23.71
45-54	$4,301.94	$26.91
55-64	$5,504.14	$36.60
65-74	$9,252.60	$63.05
75-84	$14,149.73	$78.53
85+	$23,599.77	$78.53

Abbreviations: SE, standard error.

© 2022 Lauren BN et al. *JAMA Network Open.*

eTable 2. Additive Healthcare Costs Associated with T2DM and Obesity

Age	BMI 18 – 24.9		BMI 25 – 29.9		BMI 30 – 34.9		BMI 35 – 39.9		BMI ≥ 40	
	Mean	SE	Mean	SE	Mean	SE	Mean	SE	Mean	SE
T2DM										
18-34	$4,490.41	$398.71	$5,034.16	$128.47	$5,647.21	$291.19	$7,511.58	$320.13	$7,557.97	$339.02
35-44	$4,940.23	$377.92	$5,290.70	$116.67	$6,022.37	$133.24	$6,567.07	$174.90	$7,474.51	$225.42
45-54	$4,955.90	$336.83	$5,712.67	$149.31	$5,957.37	$120.75	$6,624.00	$243.32	$8,119.70	$323.02
55-64	$5,479.51	$238.58	$5,547.47	$108.56	$5,540.05	$106.96	$6,304.53	$158.60	$7,366.53	$261.84
65-74	$4,739.47	$284.91	$4,885.80	$84.63	$5,154.62	$97.44	$5,011.37	$150.66	$5,740.25	$233.16
75-84	$3,719.79	$197.18	$3,543.80	$89.68	$3,821.32	$142.02	$3,528.34	$250.57	$3,381.39	$388.45
85+	$3,719.79	$197.18	$3,543.80	$89.68	$3,821.32	$142.02	$3,528.34	$250.57	$3,381.39	$388.45
Overweight/Obesity										
18-34	N/A	N/A	-$134.80	$0.75	$170.78	$1.50	$475.67	$6.03	$977.39	$14.73
35-44	N/A	N/A	-$188.26	$1.39	$162.94	$1.43	$631.77	$8.07	$1,476.40	$22.82
45-54	N/A	N/A	$65.98	$0.43	$549.47	$5.01	$1,093.26	$12.38	$1,766.03	$28.22
55-64	N/A	N/A	$256.55	$1.76	$873.17	$6.62	$1,324.36	$12.28	$2,326.09	$39.93
65-74	N/A	N/A	$385.14	$3.13	$1,105.90	$7.34	$1,757.97	$17.59	$2,480.15	$42.23
75-84	N/A	N/A	$403.35	$3.21	$786.75	$4.90	$1,185.64	$12.45	$1,879.85	$33.67
85+	N/A	N/A	$403.35	$3.21	$786.75	$4.90	$1,185.64	$12.45	$1,879.85	$33.67

Abbreviations: BMI, body mass index; N/A, not applicable; SE, standard error; T2DM, type 2 diabetes mellitus.

© 2022 Lauren BN et al. *JAMA Network Open.*

eTable 3. Reporting Checklist for Cost-Effectiveness Analyses from the Second Panel on Cost-Effectiveness in Health and Medicine.[9]

Element	Manuscript	Technical Supplement
**Introduction**		
Background of the problem	x	
**Study Design and Scope**		
Objectives	x	
Audience	x	
Type of analysis	x	
Target populations	x	x
Description of interventions and comparators (including no intervention, if applicable)	x	
Other intervention descriptors (e.g., care setting, model of delivery, intensity and timing of intervention)	x	
Boundaries of the analysis; defining the scope or comprehensiveness of the study (e.g., for a screening program, whether only a subset of many possible strategies are included; for a transmissible condition, the extent to which disease transmission is captured; for interventions with many possible delivery settings, whether only one or more settings are modeled)	x	
Time horizon	x	
Analytic perspectives (e.g., reference case perspectives [health care sector, societal]; other perspectives such as employer or payer)	x	
Whether this analysis meets the requirements of the reference case	x	x
Analysis plan	x	x
**Methods and Data**		
Trial-based analysis or model-based analysis. If model-based:		
Description of event pathway or model (describe condition or disease and the health states included)	x	
Diagram of event pathway or model (depicting the sequencing and possible transitions among the health states included)	x	
Description of model used (e.g., decision tree, state transition, microsimulation)	x	
Modeling assumptions	x	x
Software used	x	
Identification of key outcomes	x	
Complete information on sources of effectiveness data, cost data, and preference weights	x	x
Methods for obtaining estimates of effectiveness (including approaches used for evidence synthesis)	x	
Methods for obtaining estimates of costs and preference weights	x	x
Critique of data quality	x	

© 2022 Lauren BN et al. *JAMA Network Open.*

Element	Manuscript	Technical Supplement
**Methods and Data**		
Trial-based analysis or model-based analysis. If model-based:		
Statement of costing year (i.e., the year to which all costs have been adjusted for the analysis; e.g., 2016)	x	
Statement of method used to adjust costs for inflation	x	
Statement of type of currency	x	
Source and methods for obtaining expert judgment if applicable	N/A	N/A
Statement of discount rates	x	
**Impact Inventory**		
Full accounting of consequences within and outside the health care sector		x
**Results**		
Results of model validation	x	x
Reference case results (discounted and undiscounted): total costs and effectiveness, incremental costs and effectiveness, incremental cost-effectiveness ratios, measures of uncertainty	x	x
Disaggregated results for important categories of costs, outcomes, or both	x	x
Results of sensitivity analysis	x	x
Other estimates of uncertainty	x	x
Graphical representation of cost-effectiveness results	x	x
Graphical representation of uncertainty analyses	x	x
Aggregate cost and effectiveness information	x	x
Secondary analyses	x	x
**Disclosures**		
Statement of any potential conflicts of interest due to funding source, collaborations, or outside interests	x	
**Discussion**		
Summary of reference case results	x	
Summary of sensitivity of results to assumptions and uncertainties in the analysis	x	
Discussion of the study results in the context of results of related cost-effective analyses	x	
Discussion of ethical implications (e.g., distributive implications relating to age, disability, or other characteristics of the population)	x	
Limitations of the study	x	
Relevance of study results to specific policy questions or decisions	x	

Abbreviations: N/A, not applicable.

Notes: The table shows what components of the cost-effectiveness analysis checklist from the Second Panel on Cost-Effectiveness in Health and Medicine can be found in the analysis.

© 2022 Lauren BN et al. *JAMA Network Open.*

eTable 4. Formal Health Care Sector Impact Inventory Assessment.[9]

Type of impact	Accounted for in analysis
**Health outcomes (effects)**	
Longevity	x
Health-related-quality-of-life effects	x
Other health effects (e.g., adverse events)	x
**Medical costs**	
Paid for third-party payers	x
Paid for by patients out-out-of-pocket	x
Future related medical costs (payers and patients)	x
Future unrelated medical costs (payers and patients)	x

Notes: The table shows what components of the impact inventory assessment from the Second Panel on Cost Effectiveness Analyses in Health and Medicine are accounted for in our analysis.

© 2022 Lauren BN et al. *JAMA Network Open.*

eTable 5. Weighted NHANES and Baseline Population Characteristics.

Characteristic	NHANES	Simulated
	Mean (SE)	Mean (95%CI)[a]
Age, years	54.6 (0.6)	54.6 (54.2 – 55.0)
Female, percent	61.2% (2.3)	61.6% (60.1 – 63.4%)
Race/Ethnicity, percent[b]		
Mexican American	6.9% (1.0)	6.9% (6.1 – 7.7%)
Non-Hispanic Black	18.7% (1.8)	18.7% (17.5 – 19.9%)
Non-Hispanic White	65.2% (2.3)	65.1% (63.6 – 66.7%)
Other[c]	5.7% (1.2)	5.8% (5.2 – 6.5%)
Other Hispanic	3.5% (0.7)	3.5% (2.9 – 4.0%)
HbA1c %	7.4 (0.1)	7.4 (7.4 – 7.5)
BMI (kg/m^2)	45.8 (0.2)	45.8 (45.7 – 46.0)
Participants on insulin, percent	31.0% (2.1)	31.0% (29.4 – 32.3%)
Participants on diabetic pills, percent	77.7% (1.8)	77.6% (76.3 – 78.8%)
Baseline T2DM Severity (per IMS score), percent		
Mild	15.8% (1.7)	15.9% (14.9 – 17.0%)
Moderate	55.0% (2.6)	55.8% (54.5 – 57.2%)
Severe	29.2% (2.3)	28.3% (26.9 – 29.5%)

Abbreviations: BMI, body mass index; HbA1c, hemoglobin A1c; IMS, Individualized Metabolic Surgery; NHANES, National Health and Nutrition Examination Survey; SE, standard error; T2DM, type 2 diabetes mellitus; 95%CI, 95% credible interval.

[a] The 95%CI gives to 2.5th to 97.5th percentile of means from 1000 probabilistic simulations.
[b] Non-Hispanic Asian was added as a category in NHANES for race/ethnicity in 2011. For the sake of consistency across NHANES cycles, we did not include it here.
[c] Other includes multiracial persons and Non-Hispanic Asians

eTable 6. T2DM Remission Model Validation.

Outcome	Treatment	Model (95% credible interval)	Target (95% CI)	% in Range
T2DM Remission (% at 3 months), based on T2DM severity at baseline				
Mild	RYGB	92.9% (86.4 – 97.6%)	92.8% (88.0 – 100.0%)	92.3%
	SG	86.9% (71.9 – 96.4%)	85.2% (74.0 – 100.0%)	95.7%
Moderate	RYGB	67.6% (47.2 – 85.3%)	66.3% (60.0 – 97.0%)	79.3%
	SG	47.8% (26.6 – 70.2%)	47.2% (25.0 – 68.0%)	94.3%
Severe	RYGB	13.3% (3.6 – 25.7%)	12.8% (6.0 – 27.0%)	88.9%
	SG	5.7% (1.0 – 13.9%)	6.2% (0.0 – 12.0%)	93.7%

Abbreviations: CI, confidence interval; RYGB, Roux-en-Y gastric bypass, SG, sleeve gastrectomy; T2DM, type 2 diabetes mellitus.

© 2022 Lauren BN et al. *JAMA Network Open.*

eTable 7. Cost-Effectiveness Results Over 10-Year Time Horizon.

	Medical Therapy	SG	RYGB
**Overall**			
Mean Costs	$104,661	$118,907	$119,018
Incremental Costs (95%CI)	REF	$14,247 ($2,289 – 27,820)	$14,358 ($2,521 – 26,841)
Mean QALY	5.90	6.44	6.72
Incremental QALYs (95%CI)	REF	0.54 (0.21 – 1.13)	0.82 (0.39 – 1.60)
ICER ($/QALY gained)[a]	REF	Extendedly Dominated	$17,497
Probability preferred strategy[b]	0.0%	1.9%	98.1%
**Mild T2DM at Baseline**			
Mean Costs	$101,862	$100,393	$101,969
Incremental Costs (95%CI)	REF	-$1,469 (-$12,861 – 11,736)	$107 (-$10,812 – 12,695)
Mean QALY	6.11	6.95	7.20
Incremental QALYs (95%CI)	REF	0.84 (0.52 – 1.53)	1.09 (0.67 – 1.84)
ICER ($/QALY gained)[a]	Dominated	REF	$6,346
Probability preferred strategy[b]	0.0%	3.9%	96.1%
**Moderate T2DM at Baseline**			
Mean Costs	$104,107	$117,023	$115,659
Incremental Costs (95%CI)	REF	$12,917 (-$144 – 27,897)	$11,552 (-$1,159 – 26,435)
Mean QALY	5.90	6.55	6.87
Incremental QALYs (95%CI)	REF	0.64 (0.31 – 1.21)	0.97 (0.52 – 1.69)
ICER ($/QALY gained)[a]	REF	Dominated	$11,966
Probability preferred strategy[b]	0.0%	4.0%	96.0%
**Severe T2DM at Baseline**			
Mean Costs	$107,348	$135,333	$137,440
Incremental Costs (95%CI)	REF	$27,985 ($17,373 – 40,731)	$30,092 ($18,180 – 42,763)
Mean QALY	5.79	6.19	6.43
Incremental QALYs (95%CI)	REF	0.40 (0.11 – 1.03)	0.64 (0.20 – 1.51)
ICER ($/QALY gained)[a]	REF	Extendedly Dominated	$47,389
Probability preferred strategy[b]	11.7%	3.5%	84.8%

Abbreviations: ICER, incremental cost-effectiveness ratio; QALY, quality-adjusted life year; REF, reference group; RYGB, Roux-en-Y gastric bypass; SG, sleeve gastrectomy; T2DM, type 2 diabetes mellitus; 95%CI, 95% credible interval (i.e., 2.5th to 97.5th percentile).

[a] ICERs are calculated from the mean costs and QALYs from the 1000 probabilistic iterations and are referent to the next least costly, non-dominated strategy. Extendedly dominated indicates that the strategy gains fewer QALYs and costs more per QALY gained than another strategy, representing inefficient use of resources. Dominated indicates that the strategy results in fewer QALYs and higher costs compared with another strategy.
[b] Probability of being the preferred strategy is presented at a cost-effectiveness threshold of $100,000/QALY gained.

© 2022 Lauren BN et al. *JAMA Network Open.*

eTable 8. Cost-Effectiveness Results Over 30-Year Time Horizon

	Medical Therapy	SG	RYGB
**Overall**			
Mean Costs	$171,692	$183,045	$181,257
Incremental Costs (95%CI)	REF	$11,354 (-$2,248 – 25,771)	$9,565 (-$4,199 – 23,565)
Mean QALY	10.70	11.77	12.48
Incremental QALYs (95%CI)	REF	1.07 (0.33 – 2.29)	1.78 (0.86 – 3.37)
ICER ($/QALY gained)[a]	REF	Extendedly Dominated	$5,387
Probability preferred strategy[b]	0.0%	0.0%	100.0%
**Mild T2DM at Baseline**			
Mean Costs	$174,703	$166,797	$163,757
Incremental Costs (95%CI)	REF	-$7,906 (-$20,577 – 6,254)	-$10,946 (-$23,515 – 2,294)
Mean QALY	11.73	13.42	14.13
Incremental QALYs (95%CI)	REF	1.69 (0.95 – 3.05)	2.40 (1.46 – 4.22)
ICER ($/QALY gained)[a]	REF	Dominated	Dominant
Probability preferred strategy[b]	0.0%	0.0%	100.0%
**Moderate T2DM at Baseline**			
Mean Costs	$170,883	$182,437	$178,152
Incremental Costs (95%CI)	REF	$11,555 (-$3,115 – 27,287)	$7,269 (-$6,613 – 23,040)
Mean QALY	10.71	12.04	12.79
Incremental QALYs (95%CI)	REF	1.32 (0.58 – 2.46)	2.08 (1.12 – 3.54)
ICER ($/QALY gained)[a]	REF	Extendedly Dominated	$3,498
Probability preferred strategy[b]	0.0%	0.0%	100.0%
**Severe T2DM at Baseline**			
Mean Costs	$170,467	$202,053	$204,849
Incremental Costs (95%CI)	REF	$31,586 ($19,560 – 45,541)	$34,383 ($21,702 – 48,201)
Mean QALY	10.08	11.04	11.66
Incremental QALYs (95%CI)	REF	0.96 (0.24 – 2.21)	1.58 (0.68 – 3.27)
ICER ($/QALY gained)[a]	REF	Extendedly Dominated	$21,798
Probability preferred strategy[b]	0.2%	0.1%	99.7%

Abbreviations: ICER, incremental cost-effectiveness ratio; QALY, quality-adjusted life year; REF, reference group; RYGB, Roux-en-Y gastric bypass; SG, sleeve gastrectomy; T2DM, type 2 diabetes mellitus; 95%CI, 95% credible interval (i.e., 2.5th to 97.5th percentile).

[a] ICERs are calculated from the mean costs and QALYs from the 1000 probabilistic iterations and are referent to the next least costly, non-dominated strategy. Extendedly dominated indicates that the strategy gains fewer QALYs and costs more per QALY gained than another strategy, representing inefficient use of resources. Dominated indicates that the strategy results in fewer QALYs and higher costs compared with another strategy.
[b] Probability of being the preferred strategy is presented at a cost-effectiveness threshold of $100,000/QALY gained.

© 2022 Lauren BN et al. *JAMA Network Open.*

eFigure 1. Weight Loss Model Validation.

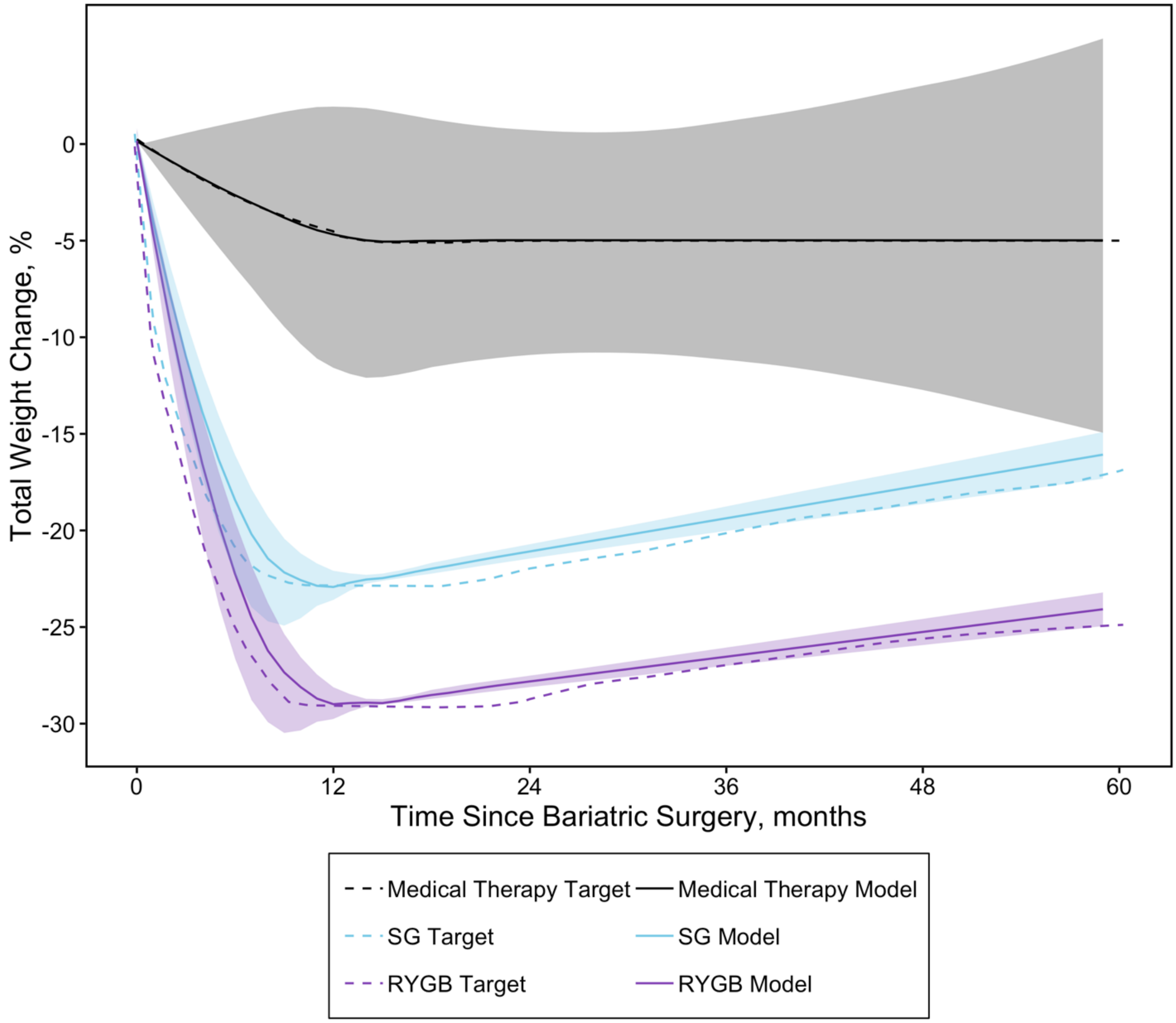

Abbreviations: RYGB; Roux-en-Y gastric bypass; SG, sleeve gastrectomy

The figure shows the model projections of the percent change in total body weight over time compared with the model validation targets. The solid lines show model output (with shaded 95% credible intervals). Dashed lines show target data from McTigue et al (2020).[10]

© 2022 Lauren BN et al. *JAMA Network Open.*

eFigure 2. T2DM Remission Over 5-Year Time Horizon.

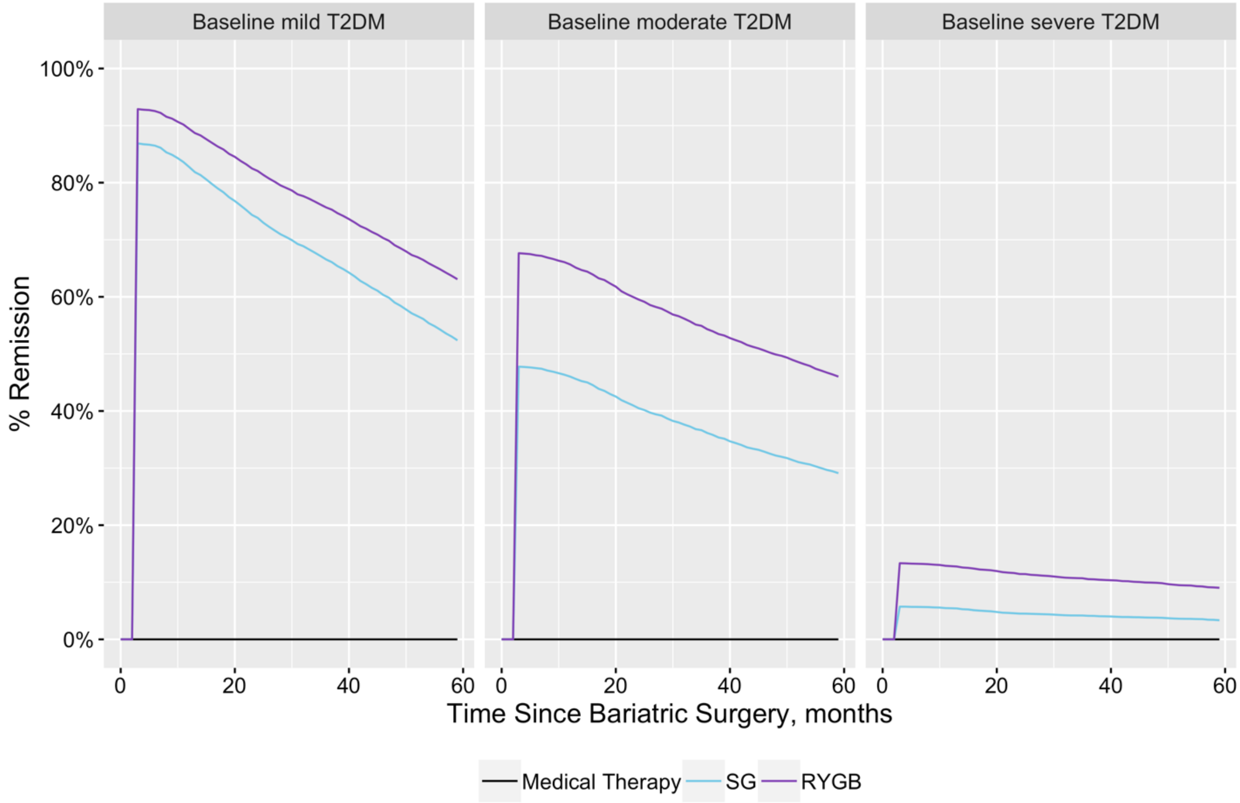

Abbreviations: RYGB, Roux-en-Y gastric bypass; SG, sleeve gastrectomy; T2DM, type 2 diabetes mellitus.

The figure shows the model projections of the proportion of individuals with remission of T2DM over time stratified by the baseline T2DM severity.

© 2022 Lauren BN et al. *JAMA Network Open.*

eFigure 3. BMI Over 30-Year Time Horizon.

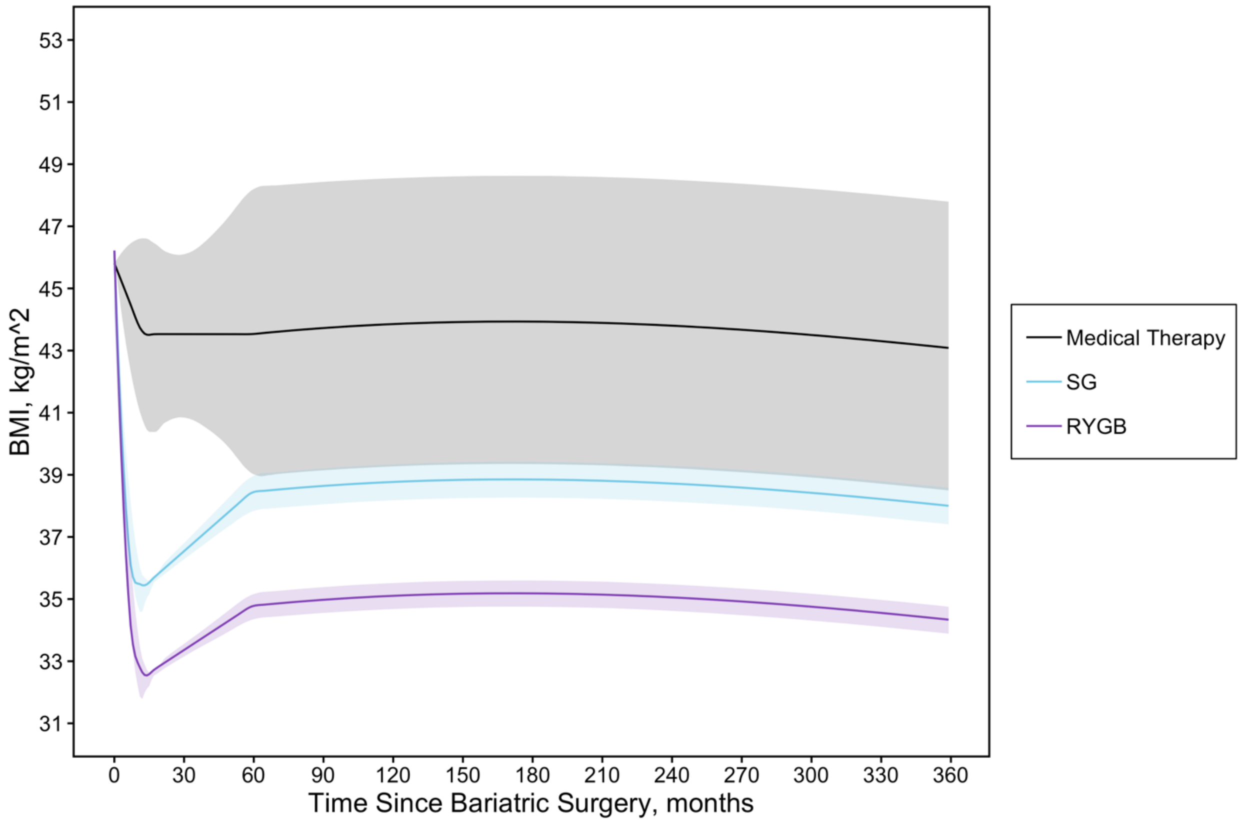

Abbreviations: BMI, body mass index; RYGB, Roux-en-Y gastric bypass; SG, sleeve gastrectomy.

The figure shows the model projections of BMI for a time horizon of up to 30 years that were used in sensitivity analysis.

© 2022 Lauren BN et al. *JAMA Network Open.*

eFigure 4. Incremental Cost-Effectiveness Scatterplot Over 5-Year Time Horizon.

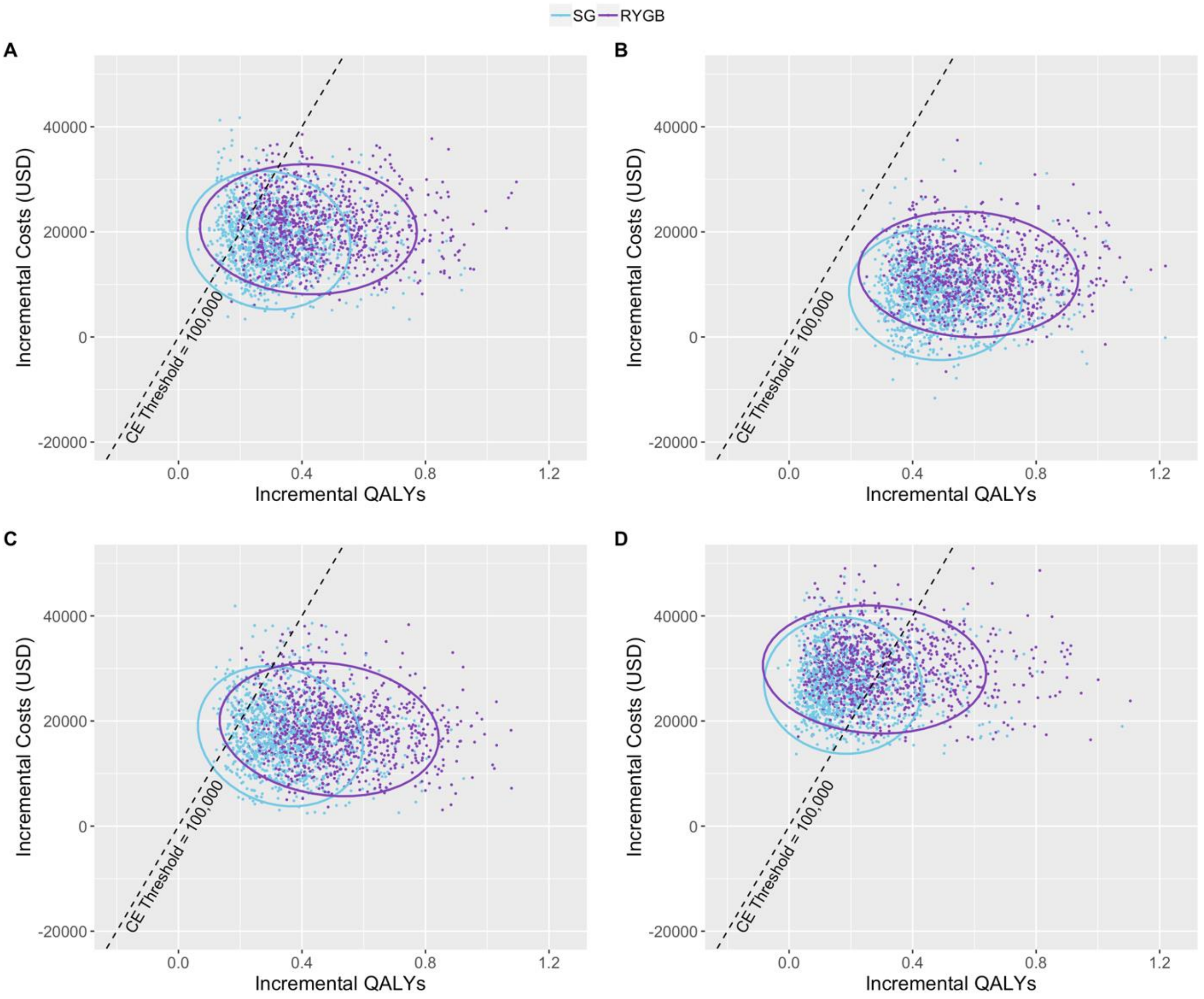

Abbreviations: CE, cost-effectiveness; QALY, Quality-adjusted life year; RYGB, Roux-en-Y gastric bypass; SG, sleeve gastrectomy; USD, United States dollar.

The figure shows the incremental costs and QALYs relative to medical therapy for each of 1000 probabilistic iterations of the model. The dashed line shows the cost-effectiveness threshold of $100,000/QALY gained; simulations below the dashed indicate the strategy is cost-effective relative to medical therapy. **Panel A** shows the results for the overall population, **Panel B** for patients with mild T2DM at baseline, **Panel C** for patients with moderate T2DM at baseline, and **Panel D** for patients with severe T2DM at baseline.

© 2022 Lauren BN et al. *JAMA Network Open.*

eFigure 5. One-Way Sensitivity Analysis Over 5-Year Time Horizon.

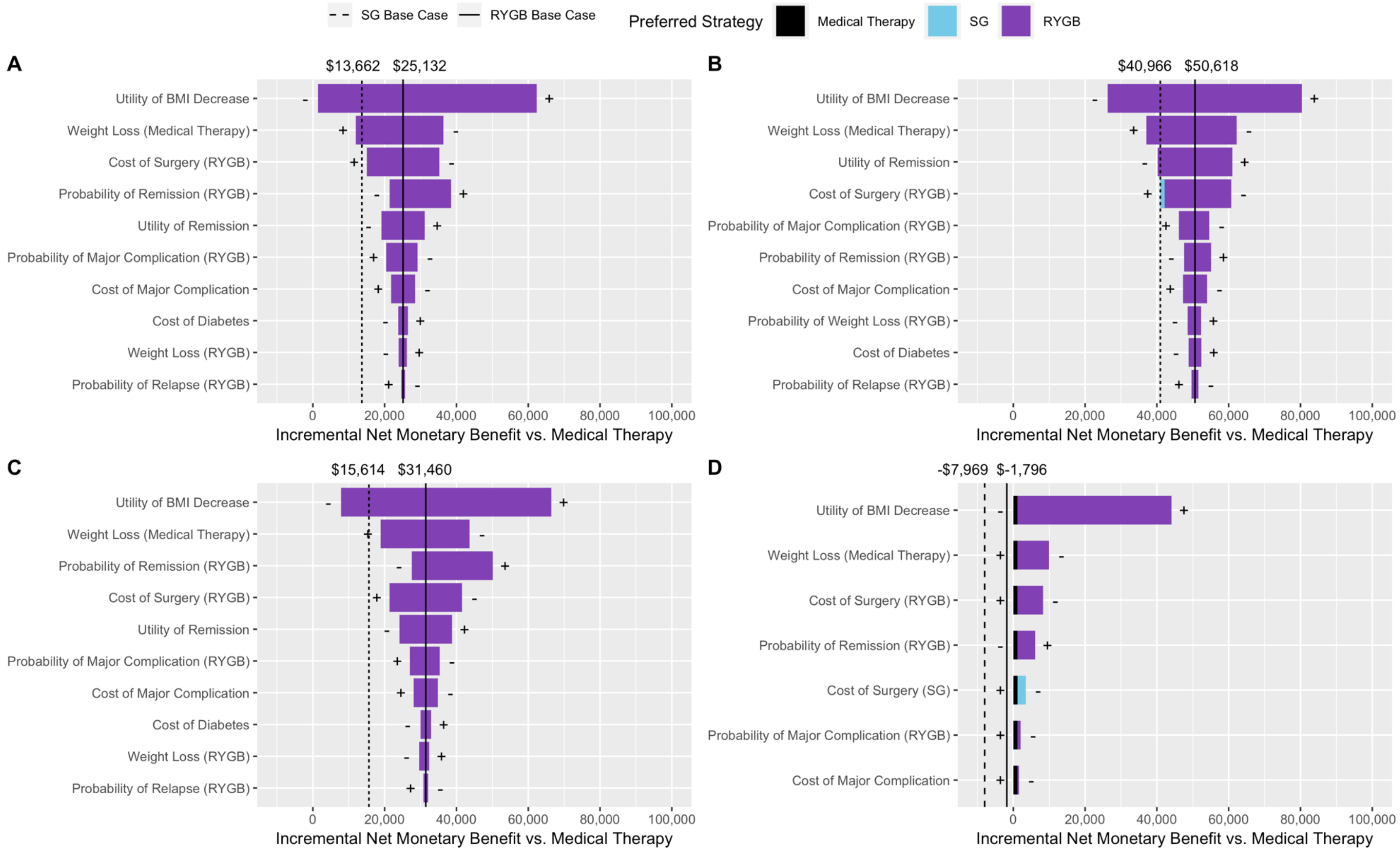

Abbreviations: BMI, body mass index; QALY, quality-adjusted life year; RYGB, Roux-en-Y gastric bypass; SG, sleeve gastrectomy; T2DM, type 2 diabetes mellitus.

The figure shows the results from the one-way sensitivity analyses in which model parameters were independently varied across a plausible range while all other parameters were held constant. **Panel A** shows the results for the overall population, **Panel B** for patients with mild T2DM at baseline, **Panel C** for patients with moderate T2DM at baseline, and **Panel D** for

© 2022 Lauren BN et al. *JAMA Network Open.*

patients with severe T2DM at baseline. The ten model parameters with the largest effect on the incremental net monetary benefit (INMB) relative to medical therapy at a cost-effectiveness threshold of $100,000 per quality-adjusted life year are shown. Only seven parameters in patients with severe T2DM resulted in medical therapy not being the preferred strategy, which is shown in **Panel D**. The horizontal bars represent the range of INMB resulting from changes in model parameter; blue bars indicate that SG was the preferred strategy and purple bars indicate that RYGB was the preferred strategy. Changes in the preferred strategy are indicated by the thick vertical lines. A "**+**" at the end of a bar denotes that the INMB occurred at the maximum value of the model parameter; a "-" denotes the INMB occurred at the minimum value of the model parameter. The base case INMB for SG and RYGB vs medical therapy are when all model inputs are set to their mean values (not probabilistic as in the primary analysis).

© 2022 Lauren BN et al. *JAMA Network Open.*

eFigure 6. Cost-Effectiveness Acceptability Curves Over 10-Year Time Horizon.

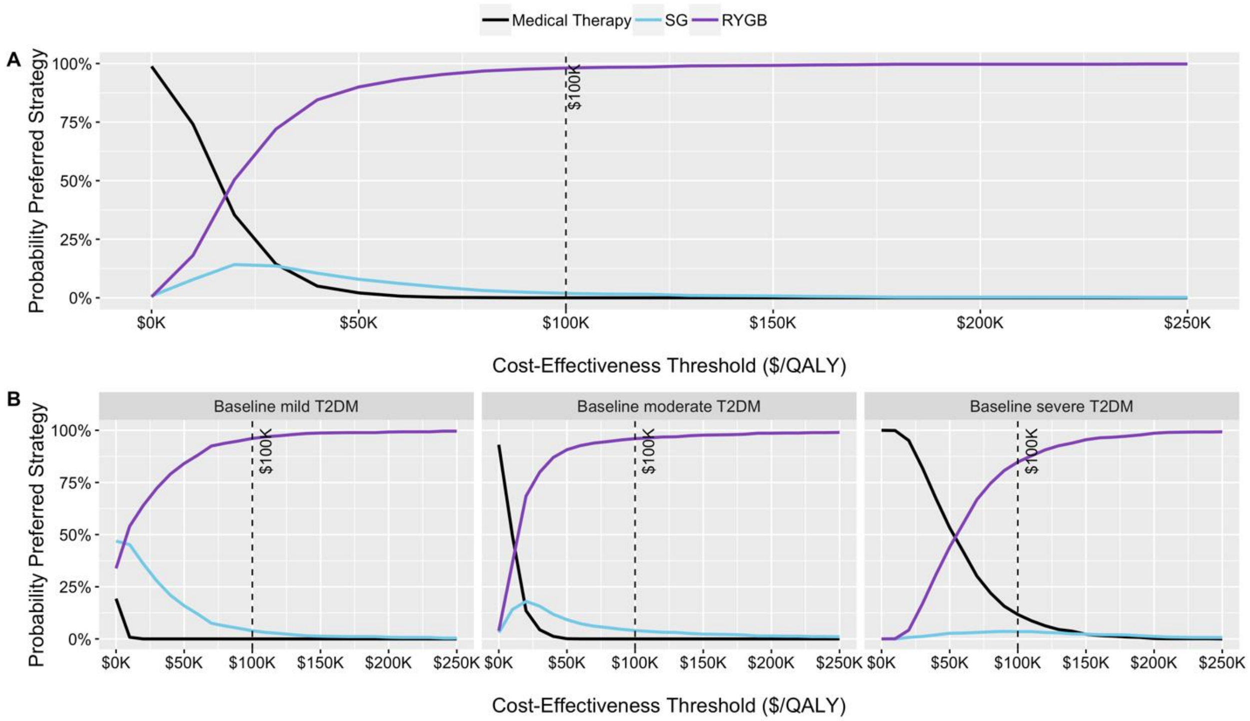

Abbreviations: QALY, quality-adjusted life year; RYGB, Roux-en-Y gastric bypass; SG, sleeve gastrectomy; T2DM; type 2 diabetes mellitus.

The figure shows the probability that each treatment was the preferred strategy across a range of cost-effectiveness thresholds from 1000 probabilistic iterations. The dashed line indicates our base-case threshold to define a strategy as cost-effective. **Panel A** shows the results in the overall population and **Panel B** shows the results stratified by baseline T2DM severity

© 2022 Lauren BN et al. *JAMA Network Open.*

eFigure 7. Cost-Effectiveness Acceptability Curves Over 30-Year Time Horizon.

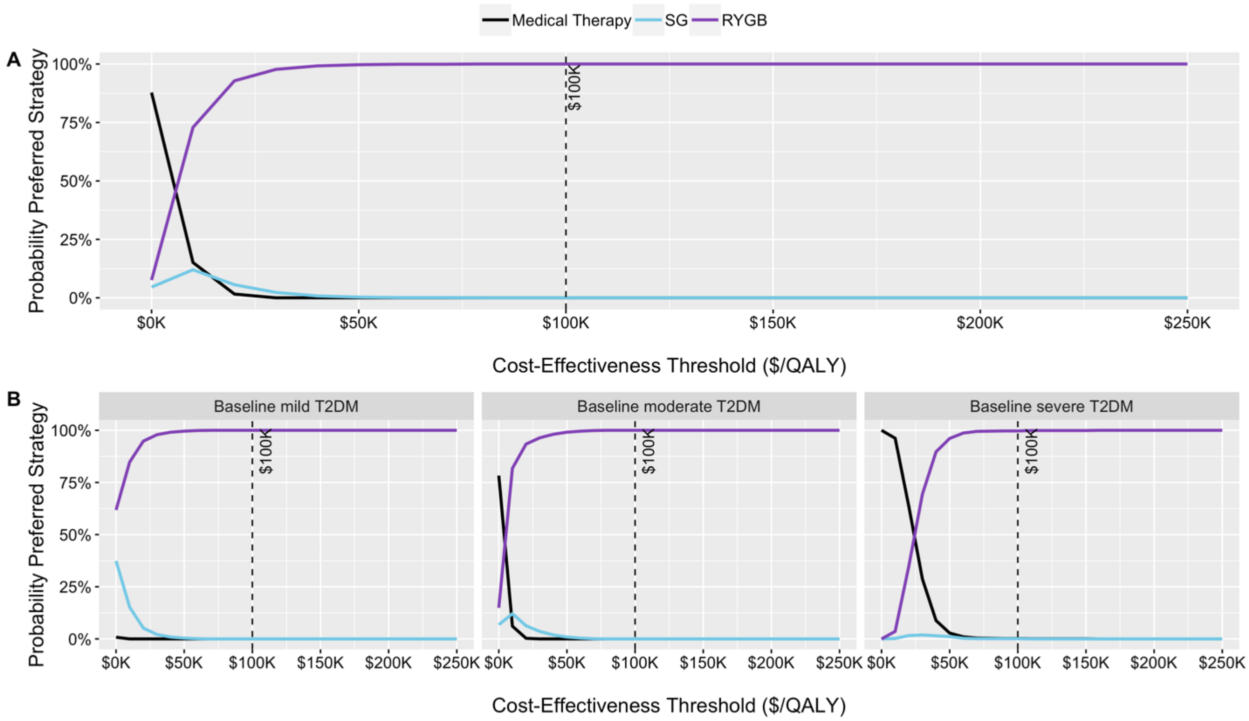

Abbreviations: QALY, quality-adjusted life year; RYGB, Roux-en-Y gastric bypass; SG, sleeve gastrectomy; T2DM; type 2 diabetes mellitus.

The figure shows the probability that each treatment was the preferred strategy across a range of cost-effectiveness thresholds from 1000 probabilistic iterations. The dashed line indicates our base-case threshold to define a strategy as cost-effective. **Panel A** shows the results in the overall population and **Panel B** shows the results stratified by baseline T2DM severity.

© 2022 Lauren BN et al. *JAMA Network Open.*

eReferences.

1. Aminian A, Brethauer SA, Andalib A, et al. Individualized Metabolic Surgery Score: Procedure Selection Based on Diabetes Severity. *Ann Surg*. 2017;266(4):650-657. doi:10.1097/SLA.0000000000002407

2. Kazi DS, Bellows BK, Baron SJ, et al. Cost-Effectiveness of Tafamidis Therapy for Transthyretin Amyloid Cardiomyopathy. *Circulation*. 2020;141(15):1214-1224. doi:10.1161/CIRCULATIONAHA.119.045093

3. Bryant KB, Moran AE, Kazi DS, et al. Cost-Effectiveness of Hypertension Treatment by Pharmacists in Black Barbershops. *Circulation*. 2021;143(24):2384-2394. doi:10.1161/CIRCULATIONAHA.120.051683

4. Medical Expenditure Panel Survey. Accessed August 12, 2019. https://meps.ahrq.gov/mepsweb/.

5. Harris-Kojetin L, Sengupta M, Park-Lee E, et al. Long-Term Care Providers and services users in the United States: data from the National Study of Long-Term Care Providers, 2013-2014. *Vital Health Stat 3*. 2016;(38):x-xii; 1-105.

6. 2010 Census Briefs: Age and Sex Composition. *US Dep Commer Econ Stat Adm U S Census Bur*. https://www.census.gov/prod/cen2010/briefs/c2010br-03.pdf.

7. Cost of Care Survey 2018. Annual Costs: National Median. Published online 2019. Accessed May 7, 2019. https://www.genworth.com/aging-and-you/finances/cost-of-care.html

8. Costs of Care. Published online 2019. Accessed May 7, 2019. https://longtermcare.acl.gov/costs-how-to-pay/costs-of-care.html

9. Sanders GD, Neumann PJ, Basu A, et al. Recommendations for Conduct, Methodological Practices, and Reporting of Cost-effectiveness Analyses: Second Panel on Cost-Effectiveness in Health and Medicine. *JAMA*. 2016;316(10):1093-1103. doi:10.1001/jama.2016.12195

10. McTigue KM, Wellman R, Nauman E, et al. Comparing the 5-Year Diabetes Outcomes of Sleeve Gastrectomy and Gastric Bypass: The National Patient-Centered Clinical Research Network (PCORNet) Bariatric Study. *JAMA Surg*. 2020;155(5):e200087-e200087. doi:10.1001/jamasurg.2020.0087

© 2022 Lauren BN et al. *JAMA Network Open.*
